# Mayer-Rokitansky-Küster-Hauser syndrome type II complicated by strangulated left inguinal ovarian-fallopian tube hernia in a pediatric patient: a case report

**DOI:** 10.1186/s12887-026-07072-2

**Published:** 2026-05-29

**Authors:** Hailong Su, Tingdong Yuan

**Affiliations:** Department of General & Pediatric Surgery, Yantai Yuhuangding Hospital Affiliated to Qingdao University, No. 20 Yuhuangding East Road, Zhifu District, Yantai, Shandong China

**Keywords:** Mayer-Rokitansky-Küster-Hauser syndrome, Inguinal hernia, Ovary, Fallopian tube, Pediatric patient

## Abstract

**Background:**

Mayer-Rokitansky-Küster-Hauser (MRKH) syndrome is a congenital disorder characterized by Müllerian duct anomalies. Patients have a significantly higher incidence of inguinal hernia (6.4%-15.6%) compared to the general female population. These hernias frequently contain ovarian or tubal tissue and are associated with a high risk of strangulation, posing a threat to future fertility. This case report details a rare pediatric presentation of MRKH syndrome type II with a strangulated inguinal ovarian-tubal hernia.

**Case presentation:**

A 7-year-5-month-old girl presented with a 28-hour history of an irreducible left inguinal mass. Ultrasound revealed a left incarcerated inguinal hernia and a right ectopic kidney. Emergency surgery confirmed a left strangulated inguinal hernia containing a necrotic ovary and fallopian tube, along with a contralateral occult hernia. Findings of uterine and vaginal agenesis with a 46,XX karyotype confirmed MRKH syndrome type II. A literature review of 24 cases (including ours) showed that inguinal hernias in MRKH are predominantly left-sided (45.8%) or bilateral (41.7%), with ovarian involvement in 87.5% of cases. In type II MRKH, renal malformations were observed in 91.7% of cases and were consistently associated with an ipsilateral inguinal hernia.

**Conclusion:**

Inguinal hernias in MRKH syndrome commonly involve gonadal tissue and carry a high incarceration risk. The presence of reproductive organs within a hernia sac or associated renal anomalies should raise strong suspicion for MRKH syndrome. Early diagnosis, timely surgical intervention to preserve viable ovarian tissue, and multidisciplinary management are crucial for protecting reproductive potential and improving long-term outcomes.

**Supplementary Information:**

The online version contains supplementary material available at 10.1186/s12887-026-07072-2.

## Background

Inguinal hernia is defined as a structural protrusion resulting from the displacement of intra-abdominal organs or tissues through a congenital or acquired defect in the inguinal region to the body surface. The lifetime risk of developing inguinal hernia ranges from 27% to 43% in males and 3% to 6% in females [1, 2]. Among subtypes, indirect inguinal hernia is the most prevalent, with typical contents including the small intestine, omentum, colon, and bladder. However, in female neonates and infants, reproductive organs—such as the ovary, fallopian tube, or uterus—are found within the hernia sac in 15% to 31% of cases, a phenomenon strongly associated with congenital anomalies of the female genital tract [3, 4]. MRKH syndrome is a rare congenital disorder characterized by Müllerian duct aplasia, leading to malformation of the female reproductive system, with an estimated incidence of 1 in 5,000 to 1 in 4,000 live female births [5]. Key clinical features include unilateral or bilateral rudimentary uterus, cervical agenesis, and complete or upper two-thirds vaginal atresia, while ovarian function, secondary sexual characteristics, and chromosomal karyotype (46,XX) remain normal. The condition is classified into type I (isolated Müllerian duct anomaly) and type II (associated with extragenital anomalies involving the urinary, skeletal, cardiac, or auditory systems). When MRKH syndrome coexists with unilateral renal agenesis or ectopic kidney and vertebral segmentation defects of the cervicothoracic spine, it is designated as Mullerian duct aplasia-Renal agenesis-Cervicothoracic Somite dysplasia (MURCS) association—a recognized subtype of type II [4, 5]. Notably, the incidence of inguinal hernia among patients with MRKH syndrome ranges from 6.4% to 15.6%, markedly exceeding that in the general female population (0.75%) [6, 7]. Despite this increased predisposition, reports of MRKH syndrome complicated by inguinal herniation of genital organs remain scarce, particularly those involving strangulation. This article presents a pediatric case of type II MRKH syndrome with left-sided strangulated inguinal ovarian-fallopian tube hernia, accompanied by a comprehensive literature review, aiming to heighten clinical awareness and diagnostic vigilance for this complex and often underrecognized condition.

## Case presentation

**Fig. 1:**
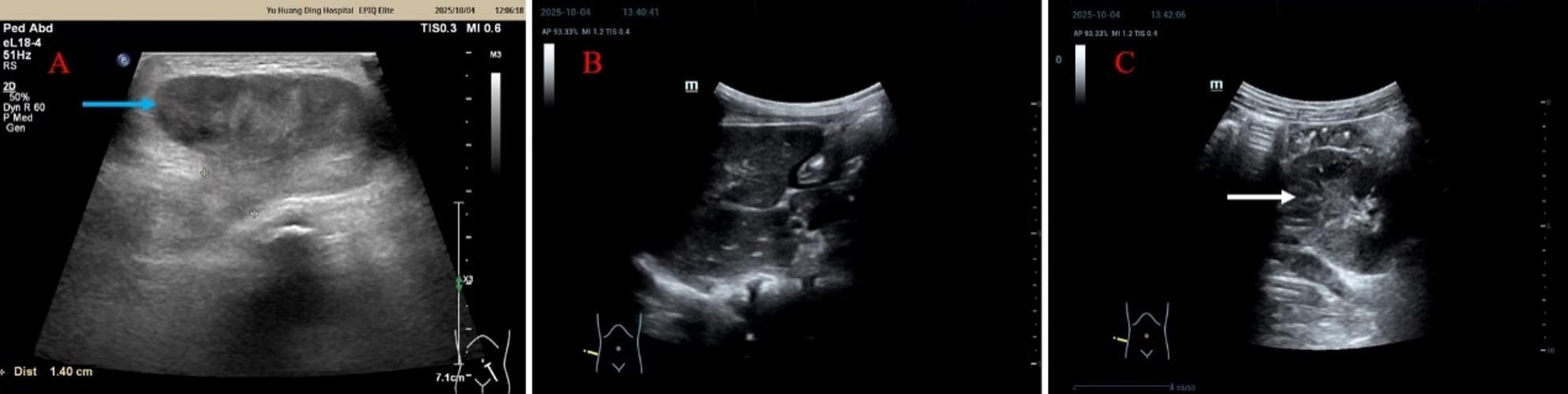
Preoperative ultrasound findings. **A** Longitudinal view of the left inguinal region demonstrates herniation of the ovary and fallopian tube (blue arrow), with continuity into the abdominal cavity. **B** Absence of renal tissue in the right renal fossa on urinary system ultrasound. **C** Transverse image of the right lower quadrant reveals an ectopic kidney with preserved renal capsule and intact collecting system (white arrow)

**Fig. 2:**
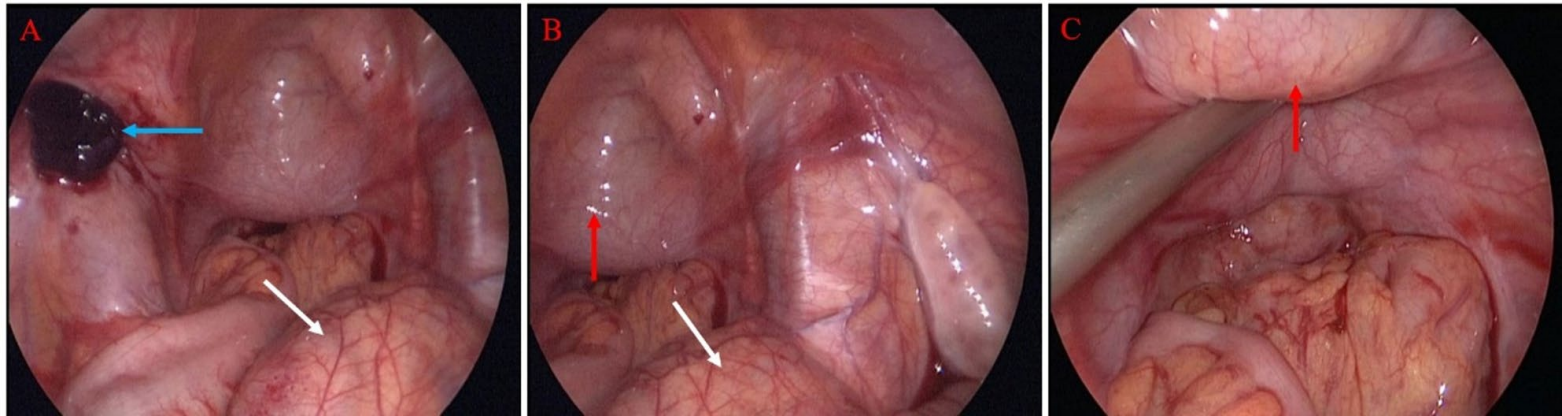
Intraoperative observations. **A** Necrotic and ischemic left ovary and fallopian tube within the hernia sac (blue arrow); retroperitoneal ectopic kidney identified on the right side (white arrow). **B** Right-sided occult inguinal hernia coexisting with ipsilateral retroperitoneal ectopic kidney (white arrow). **C** Pelvic exploration confirms absence of uterine structures; the bladder appears normal in morphology (red arrow)

**Fig. 3:**
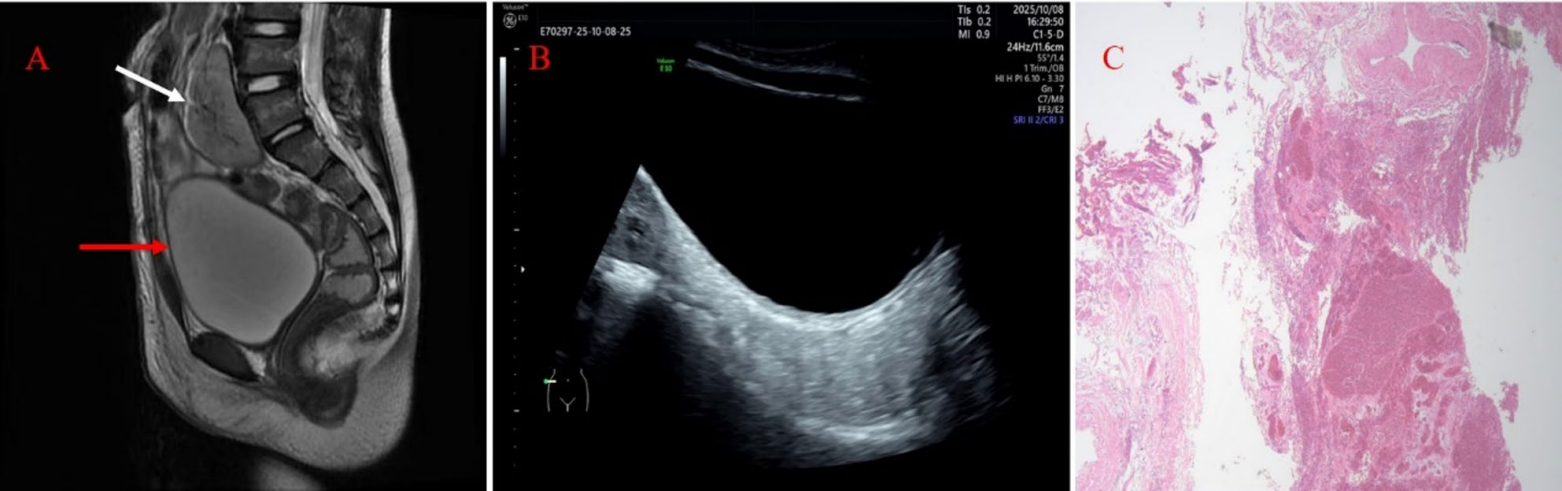
Postoperative imaging and histopathology. **A** Sagittal T2-weighted MRI of the pelvis shows a right lower abdominal ectopic kidney (white arrow) and complete absence of the uterus (red arrow indicates the bladder). **B** Gynecological ultrasound fails to detect a uterine body, and no vaginal air line is visualized. **C** Histopathological examination (H&E stain, ×4 magnification) reveals extensive hemorrhage, coagulative necrosis, and inflammatory cell infiltration in the left ovary and fallopian tube, consistent with strangulation injury

A 7-year-and-5-month-old female patient presented with a 28-hour history of a left inguinal incarcerated mass. The patient was previously healthy with no significant medical, family, psychosocial, or genetic history and no relevant prior interventions. Physical examination revealed a firm, tender mass measuring approximately 8 cm × 6 cm in the left inguinal region, irreducible by manual manipulation. Laboratory investigations demonstrated signs of acute inflammation and infection, with elevated white blood cell count (16.58 × 10⁹/L) and C-reactive protein (36.25 mg/L). Color Doppler ultrasonography of the inguinal region identified a mixed-echogenicity mass in the left inguinal area communicating with the peritoneal cavity and lacking internal blood flow, consistent with an incarcerated hernia (Fig. 1A). Renal ultrasound showed absence of renal tissue in the right renal fossa and revealed an ectopic kidney located posterior to the bowel loops in the right lower quadrant (Fig. 1B–C). The patient underwent emergency surgical exploration, which confirmed necrotic and ischemic left ovary and fallopian tube within the hernia sac (Fig. 2A), along with a contralateral occult inguinal hernia on the right side (Fig. 2B). Emergency diagnostic laparoscopy was performed to evaluate acute left inguinal symptoms. The procedure revealed a strangulated left inguinal hernia containing the ovary and fallopian tube, which was characterized by frank black discoloration and irreducibility. To assess tissue viability, the internal ring was surgically released via a left inguinal incision. The incarcerated adnexa were irrigated with warm saline and observed for 30 min. No restoration of color, turgor, or contractile response to direct tactile stimulation was observed; the tissue remained black and devoid of peristalsis—findings diagnostic of irreversible ischemic necrosis. Following documented shared decision-making with the patient’s legal guardian and urgent gynecological consultation, an open left salpingo-oophorectomy was performed through the same inguinal incision. Subsequently, laparoscopic high ligation of both indirect inguinal hernia sacs was performed at the internal ring level. Intraoperative findings further revealed absence of a uterine body in the pelvis (Fig. 2C), and a solid retroperitoneal mass on the right side (Fig. 2A–B), radiologically consistent with ectopic renal tissue. Intraoperative examination under anesthesia revealed complete absence of a visible vaginal introitus; this finding was confirmed on formal postoperative gynecologic evaluation. Postoperative abdominal magnetic resonance imaging (MRI) and gynecological ultrasound confirmed Müllerian duct agenesis, including absence of the uterus and upper vaginal segment, as well as the presence of the right ectopic kidney (Fig. 3A–B). Histopathological examination revealed extensive hemorrhage and coagulative necrosis of the left ovary and fallopian tube, confirming ischemic injury secondary to strangulated hernia (Fig. 3C). Hormonal evaluation was consistent with a prepubertal state, and chromosomal karyotyping yielded a normal 46,XX pattern. Based on these clinical, imaging, intraoperative, and laboratory findings, a definitive diagnosis of MRKH syndrome type II complicated by left inguinal ovarian-fallopian tube strangulated hernia was established. The patient recovered well postoperatively with no surgical site infections or complications.

### Literature review

A systematic literature search was conducted using the following keywords: “MRKH syndrome,” “Mayer-Rokitansky-Küster-Hauser syndrome,” “MURCS association,” “Müllerian agenesis,” “inguinal hernia,” “ovarian hernia,” “uterine hernia,” “inguinal ovary,” and “ovary.” Databases including PubMed and Web of Science were searched from their inception to November 1, 2025. Titles, abstracts, and full texts were individually reviewed to exclude review articles, duplicate reports, and non-relevant cases. A total of 21 English original studies [8–28] were ultimately included, encompassing 23 reported patients, from which clinical data were extracted. Combined with the present case, a total of 24 patients with MRKH syndrome complicated by inguinal genital organ herniation were analyzed. The median age at presentation was 20 years. Laterality analysis revealed that left-sided inguinal hernias accounted for 45.8% (11/24), bilateral hernias for 41.7% (10/24), and isolated right-sided hernias for 12.5% (3/24). Ovarian tissue was involved in 87.5% (21/24) of cases, making it the most common herniated reproductive structure. With regard to subtype classification, 12 cases were classified as type I, and 12 as type II (including 2 cases meeting criteria for MURCS association). Among type I patients, left-sided and bilateral hernias each constituted 50.0% (6/12); among type II patients, left-sided hernias represented 41.7% (5/12) and bilateral hernias 33.3% (4/12). Notably, renal malformations were observed in 91.7% (11/12) of type II patients, with a predominant involvement of the left kidney in 72.7% (8/11) of these cases. Furthermore, all 11 patients with documented inguinal hernia laterality exhibited ipsilateral renal anomalies (Table 1).

**Table 1:** Clinical characteristics of 24 patients with MRKH syndrome complicated by inguinal genital hernia

Refere-nce	Age (years)	MRKH Type	Hernia Laterality	Hernia Contents	Associated Malformations
[8]	25	I	Bilateral	Right: ovary, fallopian tube, rudimentary uterus; Left: ovary, fallopian tube	
[9]	17	II	Bilateral	Ovary bilaterally	Scoliotic deformity
[10]	36	I	Left	Rudimentary uterus	
	27	I	Left	Ovary, fallopian tube, rudimentary uterus	
	29	I	Left	Fallopian tube, rudimentary uterus	
[11]	21	I	Bilateral	Left: ovary, fallopian tube, rudimentary uterus; Right: ovary	
[12]	26	II	Right	Ovary, fallopian tube, rudimentary uterus	Cardiomegaly, right renal agenesis
[13]	20	MURCS	Right	Ovary, fallopian tube	Right renal agenesis, right lung atelectasis, cervical vertebral fusion with scoliosis
[14]	4	II	Left	Ovary, fallopian tube	Left renal agenesis
[15]	13	II	Left	Ovary, fallopian tube, rudimentary uterus	Ectopic left kidney
[16]	18	II	Left	Ovary, fallopian tube	Left renal hypoplasia
[17]	45	II	Left	Ovary, rudimentary uterus	Left renal agenesis
[18]	20	I	Bilateral	Ovary bilaterally	
[19]	20	I	Bilateral	Right: ovary, fallopian tube; Left: ovary	
[20]	10.5	MURCS	Left	Ovary	Cervical hemivertebra, left renal agenesis
[21]	31	II	Right	Ovary, fallopian tube, rudimentary uterus	Ectopic right kidney, left renal agenesis
[22]	19	I	Bilateral	Rudimentary uterus bilaterally	
[23]	12	II	Bilateral	Ovary bilaterally	Ectopic left kidney
[24]	18	I	Bilateral	Right: ovary; Left: ovary, rudimentary uterus	
[25]	20	I	Left	Ovary, fallopian tube, rudimentary uterus	
[26]	3	II	Bilateral	Ovary bilaterally	Right renal agenesis, ectopic left kidney, spinal deformities
[27]	28	I	Left	Ovary, fallopian tube, rudimentary uterus	
[28]	16	I	Left	Ovary, fallopian tube, rudimentary uterus	
Present case	7.5	II	Bilateral	Left: ovary, fallopian tube	Ectopic right kidney

## Discussion

This case presented with an incarcerated inguinal hernia as the initial clinical manifestation. Postoperative identification of a right ectopic kidney, absence of the uterus and vagina, and a 46,XX karyotype confirmed the diagnosis of MRKH syndrome type II. Consistent with existing literature, the incidence of inguinal hernia in patients with MRKH syndrome is markedly higher than in the general female pediatric population, with hernia contents frequently comprising the ovary or remnants of Müllerian duct derivatives [3]. The underlying pathological mechanism stems from the characteristic anatomical anomalies associated with MRKH syndrome. Specifically, the uterus is typically absent or rudimentary, and the round ligament attaches distally to the inguinal region and proximally to a hypoplastic uterine horn. Together with the adnexa connected via the proper ovarian ligament, this forms a cord-like structure that extends toward the inguinal canal—functionally analogous to the male gubernaculum testis [13]. When combined with a patent processus vaginalis, increased intra-abdominal pressure can drive the herniation of the rudimentary uterine horn or adnexal structures along this pathway, mimicking testicular descent and predisposing to incarceration [10].

From an embryological standpoint, MRKH syndrome and inguinal genital organ herniation share a common developmental origin. Abnormal development of the paramesonephric (Müllerian) ducts not only disrupts urogenital tract formation but may also alter the structural integrity of the inguinal region [29]. This association is particularly evident in type II MRKH syndrome, where renal malformations are highly prevalent (93.3%), predominantly affecting the left kidney (78.6%). Notably, in 85.7% of these cases, the renal anomaly is ipsilateral to the hernia, suggesting that urinary system maldevelopment may contribute to localized abdominal wall weakness through altered anatomical relationships. However, the present case—featuring a right-sided ectopic kidney contralateral to a left-sided hernia—highlights that while such patterns exist, individual variation necessitates careful clinical evaluation beyond assumed correlations.

Our literature review confirms a distinct lateral distribution pattern: left-sided (48.1%) and bilateral (37.0%) inguinal hernias predominate, whereas right-sided hernias are relatively rare (14.8%). This left-sided predominance is even more pronounced among type II patients (63.6%), contrasting sharply with the typical right-side predominance observed in sporadic inguinal hernias in the general population [3, 10]. We propose that this laterality bias may be influenced by the high frequency of ipsilateral renal anomalies and potentially greater susceptibility of the left Müllerian-derived structures to developmental perturbations, leading to compromised support in the left inguinal region.

MRKH syndrome is most commonly diagnosed during adolescence following evaluation for primary amenorrhea or chronic pelvic pain, with a median age at presentation of 20 years among the 27 reviewed cases. The early diagnosis in this 7.5-year-old child—prompted by an acute incarcerated hernia—is exceptionally rare, as Müllerian duct anomalies are typically asymptomatic before puberty [19]. In young pediatric patients, transient increases in intra-abdominal pressure due to crying, coughing, or physical activity can readily precipitate herniation of mobile genital organs into the inguinal canal, increasing the risk of incarceration. Delayed recognition of an incarcerated ovarian hernia carries a significant risk of torsion and necrosis, reported to occur in 2%–33% of cases [3]. Therefore, timely diagnosis is critical for preserving ovarian endocrine and potential reproductive function.

A common diagnostic pitfall is the misclassification of such presentations as simple indirect inguinal hernias, thereby overlooking the underlying congenital genital anomaly. Imaging plays a pivotal role in accurate diagnosis. Pelvic ultrasound and magnetic resonance imaging (MRI) demonstrate high sensitivity and specificity in identifying uterine agenesis, ectopic ovarian positioning, and associated renal abnormalities. Given these findings, we strongly recommend routine pelvic imaging—including transabdominal or transperineal gynecologic ultrasound—in all female children presenting with inguinal hernia, especially when concurrent urinary tract anomalies are present. Such proactive screening enables earlier detection of Müllerian duct anomalies and facilitates timely multidisciplinary management involving pediatric surgery, urology, and adolescent gynecology.

The surgical management of inguinal hernia in female children must account for the possibility that reproductive organs—particularly the ovary and fallopian tube—are contained within the hernia sac. These hernias carry a high risk of strangulation, which may compromise future fertility and hormonal function [3]. Preoperative identification of ovarian tissue within the hernia sac on ultrasound should raise clinical suspicion for an underlying Müllerian duct anomaly and warrant comprehensive pelvic imaging. Furthermore, the presence of concomitant urinary or skeletal malformations should prompt systematic gynecologic assessment to detect MRKH syndrome at an early stage. Conversely, in patients already diagnosed with MRKH syndrome, routine ultrasound surveillance of the inguinal regions is advisable. If an occult hernia is detected, prophylactic surgical intervention—such as high ligation of the hernia sac—should be considered even in the absence of symptoms, to prevent acute incarceration and preserve gonadal viability.

For symptomatic hernias, the choice between open and laparoscopic repair should be individualized based on patient factors and surgeon expertise. Intraoperative assessment of the viability of herniated tissues is essential; viable ovaries and fallopian tubes should be carefully reduced into the peritoneal cavity without resection. Unfortunately, in this case, the patient required salpingo-oophorectomy due to irreversible ischemic necrosis of the incarcerated adnexa. This outcome underscores the consequences of delayed diagnosis and highlights the importance of early recognition and intervention. With timely management, fertility-preserving outcomes are achievable. Although this case suggests a mechanistically plausible association between MRKH-related anatomical features and inguinal herniation, its generalizability is limited by the inherent constraints of the case report format and the absence of longitudinal data on gonadal function. The parents expressed relief that the underlying condition had been identified despite loss of the affected ovary and emphasized the importance of early screening for other children.

## Conclusion

In summary, patients with MRKH syndrome are at significantly increased risk of inguinal hernia, characterized by a left-sided predominance, frequent involvement of the ovary and fallopian tube, and a high propensity for incarceration. This clinical phenotype arises directly from the syndrome’s unique anatomical features, including the aberrant trajectory of the round ligament and persistence of the processus vaginalis. Clinicians should maintain a high index of suspicion for MRKH syndrome in any female child presenting with an inguinal hernia, particularly in the context of coexisting urinary system malformations. Early diagnosis through routine pelvic imaging is crucial, as delayed treatment of an incarcerated ovarian hernia can rapidly result in irreversible gonadal necrosis, compromising both endocrine function and future reproductive potential. Implementing standardized screening protocols and promoting interdisciplinary collaboration are essential steps toward improving long-term outcomes. Ultimately, optimizing care requires heightened clinical awareness, proactive surveillance, fertility-sparing surgical strategies, and coordinated, lifelong multidisciplinary follow-up.

## Supplementary Information

Supplementary Material 1.

## Data Availability

All data generated or analysed during this study are included in the main text.

## Abbreviations

MRKH Mayer: Rokitansky-Küster-Hauser
MURCS Mullerian duct aplasia: Renal agenesis-Cervicothoracic Somite dysplasia
