# Supplementary material for: Mayer-Rokitansky-Küster-Hauser syndrome type II complicated by strangulated left inguinal ovarian-fallopian tube hernia in a pediatric patient: a case report
Source: BMC Pediatr. 2026 May 29;26:696. doi: 10.1186/s12887-026-07072-2 (PMC13425783; doi:10.1186/s12887-026-07072-2)
Supplement: Supplementary file 1 — Supplementary Material 1. [file 12887_2026_7072_MOESM1_ESM.pdf]

CARE Checklist of information to include when writing a case report

CARE case report guidelines

(cc) BY-NC-ND

Topic	Item	Checklist item description	Reported on Line
Title	1	The diagnosis or intervention of primary focus followed by the words "case report"	Lines 1-2
Key Words	2	2 to 5 key words that identify diagnoses or interventions in this case report, including "case report"	Lines 34-35
Abstract (no references)	3a	Introduction: What is unique about this case and what does it add to the scientific literature?	Lines 10-17
	3b	Main symptoms and/or important clinical findings	Lines 18-22
	3c	The main diagnoses, therapeutic interventions, and outcomes	Lines 22-27
	3d	Conclusion—What is the main "take-away" lesson(s) from this case?	Lines 28-33
Introduction	4	One or two paragraphs summarizing why this case is unique (**may include references**)	Lines 36-64
Patient Information	5a	De-identified patient specific information.	Line 66
	5b	Primary concerns and symptoms of the patient.	Lines 66-67
	5c	Medical, family, and psycho-social history including relevant genetic information	Lines 67-68
	5d	Relevant past interventions with outcomes	Lines 67-68
Clinical Findings	6	Describe significant physical examination (PE) and important clinical findings.	Lines 69-70
Timeline	7	Historical and current information from this episode of care organized as a timeline	Lines 69-70,lines 72-77
Diagnostic Assessment	8a	Diagnostic testing (such as PE, laboratory testing, imaging, surveys).	Lines 70-77
	8b	Diagnostic challenges (such as access to testing, financial, or cultural)	Lines 75-77
	8c	Diagnosis (including other diagnoses considered)	Lines 102-106
	8d	Prognosis (such as staging in oncology) where applicable	N/A
Therapeutic Intervention	9a	Types of therapeutic intervention (such as pharmacologic, surgical, preventive, self-care)	Lines 88-92
	9b	Administration of therapeutic intervention (such as dosage, strength, duration)	Lines 81-88
	9c	Changes in therapeutic intervention (with rationale)	Lines 88-92
Follow-up and Outcomes	10a	Clinician and patient-assessed outcomes (if available)	Lines 106-107
	10b	Important follow-up diagnostic and other test results	Lines 96-101
	10c	Intervention adherence and tolerability (How was this assessed?)	Lines 80-81
	10d	Adverse and unanticipated events	Lines 83-88
Discussion	11a	A scientific discussion of the strengths AND limitations associated with this case report	Lines 233-236
	11b	Discussion of the relevant medical literature **with references**.	Lines 157-200,lines 214-215
	11c	The scientific rationale for any conclusions (including assessment of possible causes)	Lines 160-172,lines 181-185
	11d	The primary "take-away" lessons of this case report (without references) in a one paragraph conclusion	Lines 239-254
Patient Perspective	12	The patient should share their perspective in one to two paragraphs on the treatment(s) they received	Lines 236-238
Informed Consent	13	Did the patient give informed consent? Please provide if requested	Yes ☑ No ☐
